# microRNA-Based Biomarkers in Alzheimer’s Disease (AD)

**DOI:** 10.3389/fnins.2020.585432

**Published:** 2020-10-30

**Authors:** Yuhai Zhao, Vivian Jaber, Peter N. Alexandrov, Andrea Vergallo, Simone Lista, Harald Hampel, Walter J. Lukiw

**Affiliations:** ^1^LSU Neuroscience Center, Louisiana State University Health Sciences Center, New Orleans, LA, United States; ^2^Department of Cell Biology and Anatomy, Louisiana State University Health Science Center, New Orleans, LA, United States; ^3^Russian Academy of Medical Sciences, Moscow, Russia; ^4^Sorbonne University, GRC n° 21, Alzheimer Precision Medicine (APM), AP-HP, Pitié-Salpêtrière Hospital, Paris, France; ^5^Brain & Spine Institute (ICM), INSERM U 1127, CNRS UMR 7225, Boulevard de l’Hôpital, Paris, France; ^6^Institute of Memory and Alzheimer’s Disease (IM2A), Department of Neurology, Pitié-Salpêtrière Hospital, AP-HP, Boulevard de l’hôpital, Paris, France; ^7^Department of Ophthalmology, LSU Neuroscience Center Louisiana State University Health Science Center, New Orleans, LA, United States; ^8^Department of Neurology, LSU Neuroscience Center Louisiana State University Health Science Center, New Orleans, LA, United States

**Keywords:** aging, Alzheimer’s disease, AD biomarkers, AD diagnostics, AD heterogeneity, human biochemical individuality, inflammatory neurodegeneration, microRNA (miRNA)

## Abstract

Alzheimer’s disease (AD) is a multifactorial, age-related neurological disease characterized by complex pathophysiological dynamics taking place at multiple biological levels, including molecular, genetic, epigenetic, cellular and large-scale brain networks. These alterations account for multiple pathophysiological mechanisms such as brain protein accumulation, neuroinflammatory/neuro-immune processes, synaptic dysfunction, and neurodegeneration that eventually lead to cognitive and behavioral decline. Alterations in microRNA (miRNA) signaling have been implicated in the epigenetics and molecular genetics of all neurobiological processes associated with AD pathophysiology. These changes encompass altered miRNA abundance, speciation and complexity in anatomical regions of the CNS targeted by the disease, including modified miRNA expression patterns in brain tissues, the systemic circulation, the extracellular fluid (ECF) and the cerebrospinal fluid (CSF). miRNAs have been investigated as candidate biomarkers for AD diagnosis, disease prediction, prognosis and therapeutic purposes because of their involvement in multiple brain signaling pathways in both health and disease. In this review we will: (i) highlight the significantly heterogeneous nature of miRNA expression and complexity in AD tissues and biofluids; (ii) address how information may be extracted from these data to be used as a diagnostic, prognostic and/or screening tools across the entire continuum of AD, from the preclinical stage, through the prodromal, i.e., mild cognitive impairment (MCI) phase all the way to clinically overt dementia; and (iii) consider how specific miRNA expression patterns could be categorized using miRNA reporters that span AD pathophysiological initiation and disease progression.

## Overview

Alzheimer’s disease (AD) represents a complex, multifactorial, age- and gender-related, progressive neurological degeneration of the human brain and central nervous system (CNS) whose clinical course is highly variable, heterogeneous, extremely insidious, and ultimately lethal. Final outcomes involve complex and irreversible alterations in behavior, inability to conduct daily activities, visual, visuospatial and perceptive disruption, and escalating deficits in cognition and impairment of recent memories in the AD patient, while older memories are often retained. Symptomology for AD is also highly variable, interactive and progressive to the extent of daily to weekly changes in the AD patient’s psychiatric condition. Clinically, toward the termination of the AD process there is usually progressive forfeiture of the swallowing reflex (dysphagia) and the onset of inspirational pneumonia to which most AD patients succumb over a clinical course averaging about ∼5–12 years (Dinsmore, 1999; Ahluwalia and Vellas, 2003; DeTure and Dickson, 2019; von Arnim et al., 2019; Cao et al., 2020^1^,^2^,^3^; last accessed 26 August 2020). The definitive diagnosis of AD is one of the most difficult and challenging in neurology (Arvanitakis et al., 2019; Fierini, 2020; Ghaffari et al., 2020; Guest et al., 2020; Habes et al., 2020; Turner et al., 2020). AD is more often than not accompanied by other multimodal dementing neuropathologies including neurovascular and/or cardiovascular disease involving vascular-based dementia, multiple infarct dementia (MID) and/or strokes or “mini-strokes,” frontotemporal dementia (FTD), hippocampal sclerosis, Lewy body disease, and several other dementing illnesses and comorbidities such as Down’s syndrome (trisomy 21), epilepsy and prion disease [including bovine spongiform encephalopathy (BSE; mad cow disease), Creutzfeldt–Jakob disease, Gerstmann–Sträussler–Scheinker syndrome, and other relatively rare human prion disorders] and other rare AD subtypes (Dinsmore, 1999; Lemcke and David, 2018; DeTure and Dickson, 2019; Checksfield, 2020; Fierini, 2020; Emrani et al., 2020; Habes et al., 2020; Williams et al., 2020^4^ ; last accessed 26 August 2020). The accurate identification of AD is exacerbated by the global lack of routine diagnostic tools for identifying patients early enough in their disease course, i.e., the “prodromal” period, for designing a suitable intervention or prospective treatment regimen. *Of equal concern is our lack of basic understanding of the underlying root causes of AD and the widely observed variability in the clinical presentation of AD once the onset of the disease process is clinically recognized* (Ashford et al., 1992; Hudon et al., 2020; Patnode et al., 2020). For AD only symptomatic treatments that suppress the clinical manifestations are currently available (Lukiw et al., 2012; Hampel and Lista, 2013; Lukiw, 2013a,b; Praticò, 2013; Hampel et al., 2014; Kim et al., 2014; Yanagida et al., 2017; Blennow and Zetterberg, 2018; Cole and Seabrook, 2020). Increasing stratification and categorization of AD, comorbidities and inter-current illness that may have contributed to the clinical diagnosis and outcome of AD may be required to develop the most efficacious treatments (see below).

## Clinical Aspects of AD Heterogeneity

With regard to the overall general classification of AD, affected patients are broadly categorized as having either an early onset (EOAD, under ∼65 years of age), or late onset (LOAD, over ∼65 years of age); about ∼5% of all AD cases appear to have a genetic component (see below) while the remaining ∼95% of all AD cases are of an idiopathic or sporadic nature, or are of an unknown origin (Guerreiro et al., 2012; Jiang et al., 2013; Barnes et al., 2015; Cao et al., 2020; Dumurgier and Tzourio, 2020). Over 50 susceptibility genes and gene loci have been associated with LOAD (Sims et al., 2020). The transmissibility of AD amongst *Homo sapiens* by casual or iatrogenic routes and involving self-propagating amyloidogenic prion-like lipoprotein aggregates or free or microvesicle-encapsulated pathogenic miRNAs has not been completely ruled out (Lukiw et al., 2012; Burwinkel et al., 2018; Lemcke and David, 2018; Caughey and Kraus, 2019; Hampton, 2019; Lukiw, 2020a,b; Lukiw and Pogue, 2020).

**FIGURE 1:**
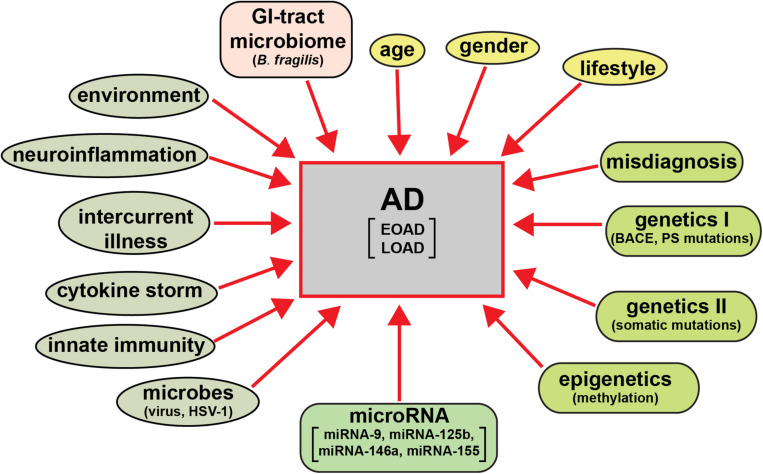
AD is an extremely heterogeneous neurological disorder. Just as there are multiple factors that have significant potential to contribute to AD type and AD symptomatic and molecular-genetic heterogeneity, the neuropathological pathways and pathway biomarkers encompassing these factors can be intercepted and interrogated to obtain extremely useful diagnostic information which more clearly define the prodrome, onset and course of AD as well as related progressive neurodegenerative disorders. The pervasive and modulatory nature of miRNAs in both brain tissues and multiple biofluid compartments (such as the ECF, CSF, and blood serum) make miRNAs ideal candidates to expand our understanding of the diagnosis of temporal aspects of the AD pathophysiology.

The clinical analysis and categorization of presenile dementia in the elderly typically incorporates semi-structured interviews with AD patients, their care-givers and other informants to obtain information necessary to rate the individual’s cognitive performance in six domains of cognitive and functional performance: memory skills, orientation, judgment and problem solving, community affairs, home and hobbies, and personal care; the clinical dementia rating (CDR; a five point scale ranging from 0 or normal to 3 for severe dementia) protocol has evolved as a condensed, useful, reliable and valid global assessment measure for AD (Morris, 1997; Verhülsdonk et al., 2015^5^). There are in addition several other widely used psychiatric and diagnostic tests including the mini-mental status exam (MMSE; Arvanitakis et al., 2019; Alzheimer’s Association, 2020). While the diagnostic criteria for AD vary globally systematic review and meta-analysis of AD incidence and prevalence have repeatedly revealed several globalized trends (Zhu et al., 2019; Dumurgier and Tzourio, 2020). These include the significantly higher occurrence of AD in aged human females (about ∼2 times greater than that in males) and strong association with aging (Guerreiro et al., 2012; Bhattacharjee and Lukiw, 2013; Jiang et al., 2013; Cao et al., 2020; Dumurgier and Tzourio, 2020; Lukiw, 2020a,b). The wide variety of behavioral disorders, extreme heterogeneity in neurological disturbances, mnemonic and cognitive deficits including significant age- and gender-based differences, combined with supplementary neurological diseases such as neurovascular disease and ischemic and/or hemorrhagic stroke are extremely common, especially in the most elderly of LOAD patients (Dinsmore, 1999; Fierini, 2020; Emrani et al., 2020; Habes et al., 2020). Environmental factors and life style triggers such as occupational exposures to pesticides, organic solvents, environmental neurotoxins such as aluminum and mercury, anesthetics and/or food additives, education, smoking, increased body-mass index (BMI), obesity, metabolic syndrome and diabetes, microbial and gastrointestinal (GI) tract microbiome contributions such as highly proinflammatory *Bacteroides fragilis* lipopolysaccharides (BF-LPS) to the onset and development of AD are being increasingly recognized but their mechanism of pathological contribution are in most cases not well understood, and are currently under intense research investigation (Hill et al., 2014a,b; Altveş et al., 2020; Lukiw, 2020a,b; Rahman et al., 2020). *A priori* this strongly suggests: (i) that a considerable array of factors have been evidenced to contribute to the onset and propagation of AD; and (ii) that a very wide range of molecular-genetic, neurophysiological and neurobiological mechanisms, pathways and signaling processes are affected in the AD brain (Figure 1).

## Traditional Pathophysiological Biomarkers for AD Are Non-Specific

Alzheimer’s disease neuropathology encompasses several interrelated features: (i) the progressive disorganization and dropout of neocortical synapses that involve loss of selective synaptic components, synaptic atrophy, “pruning” and synaptic loss; (ii) neuronal atrophy, cytoarchitectural deficits and neurite retraction and degeneration, neuronal cell death and loss of inter-neuronal communication; (iii) the progressive deposition and accumulation of amyloid-beta (Aβ) peptides and other insoluble end-stage oxidized lipoproteins into dense, pro-inflammatory senile plaque (SP) aggregates; (iv) the accumulation and aggregation of hyper-phosphorylated tau proteins into neurofibrillary tangles (NFT) that disrupt the normal neural cell cytoarchitecture; (v) neurovascular pathology; (vi) dysfunctional autophagy; (vii) progressive inflammatory neurodegeneration and anatomical targeting of specific anatomical regions of the brain and primarily the association neocortex and hippocampal CA1 regions; (viii) alterations in the innate-immune response and other immunological biomarkers; (ix) changes in the gastrointestinal tract (GI-tract) microbiome; (x) dysfunction and alterations in the glymphatic system of the CNS; (xi) multiple functional associations with diet, obesity, metabolic disease and diabetes; and (xii) blood lipoprotein composition and blood type (Lukiw et al., 1992; Lukiw, 2007; Cogswell et al., 2008; Lukiw, 2013a,b; Praticò, 2013; Hampel and Lista, 2013; Kim et al., 2014; Sherva et al., 2014; Canobbio et al., 2015; De Marco and Venneri, 2015; Jin et al., 2015; Verhülsdonk et al., 2015; Wang et al., 2015; Zhao et al., 2015; Blennow and Zetterberg, 2018; Hampel et al., 2018a,b,c; Arvanitakis et al., 2019; von Arnim et al., 2019; Cole and Seabrook, 2020; Dumurgier and Tzourio, 2020; Hampel et al., 2020a,b; Khoury and Grossberg, 2020; Lewczuk et al., 2020; McGurran et al., 2020; Rodriguez and Lachmann, 2020; Rossini et al., 2020; Stanciu et al., 2020; Turner et al., 2020).

These highly interactive characteristics once again collectively underscore the participation of multiple pathogenic pathways, and the involvement of multiple deficits in the expression of CNS genes and genetic regulatory mechanisms in AD (Colangelo et al., 2002; Jaber et al., 2019; Sims et al., 2020). Importantly, the magnitude of each of these neuropathological biomarkers varies widely amongst the prodromal, moderate, and severe states of AD and not one of these multiple features of AD change are either characteristic or distinguishing for the AD phenotype. Put another way many of these attributes are in part typical of other incapacitating age-related neurological disorders of the human CNS. Accordingly, this culminates in a remarkably heterogeneous neuropathological framework for AD, with significant variations in disease initiation, onset, progression, severity of neuropathology, extent of behavioral disruption, cognitive deficits and memory loss, visual and visuospatial impairment, time course and other temporal aspects, CDR or MMSE ratings in individual AD patients and the frequent contribution of inter-current illness, and particularly with other kinds of age-related neurological disease (Verhülsdonk et al., 2015; Rossini et al., 2020; Tetreault et al., 2020). Given the enormous complexity of traditional biomarkers and their compartmentalization in AD onset, course and diagnosis, it is clear that the most informative molecular biomarkers for AD will be those which are involved in multiple regulatory aspects of brain function and neuropathological signaling. The participation of small families of pathology-implicated miRNAs are emerging as prime candidates that help define the molecular genetics and epigenetics of AD.

### microRNA (miRNA) - Definitions and Actions

**FIGURE 2:**
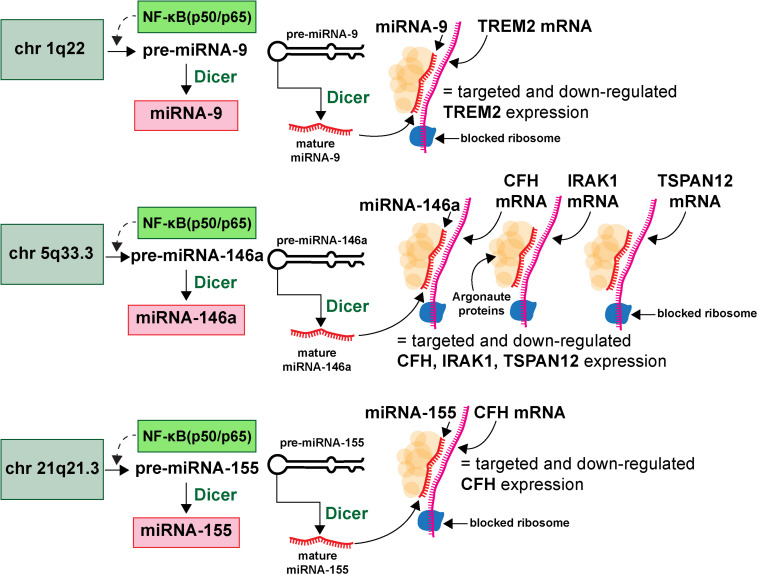
Highly simplified schematic of an AD-relevant microRNA-messenger RNA (miRNA-mRNA) regulatory network involving 3 different miRNAs and 4 different mRNAs; this drawing graphically illustrates the molecular-genetic mechanism of microRNA (miRNA) generation and targeted miRNA-mRNA interaction; selective pathogenic families of miRNAs (for example miRNA-9, miRNA-146a and miRNA-155), transcribed from genes located on 3 different chromosomes (chr 1q22, chr 5q33.3, chr 21q21.3) generate precursor miRNAs (pre-miRNAs) which are subsequently processed into neurologically active mature miRNAs (miRNA-9, miRNA-146a and miRNA-155 shown as an example); many more chromosomes, miRNAs and mRNAs and miRNA-mRNA signaling networks are probably involved; many AD-relevant miRNA encoding genes are under transcriptional control by the pro-inflammatory transcription factor NF-kB (p50/p65); mature miRNAs subsequently find their target mRNAs (TREM2, CFH, IRAK-1, and TSPAN12 shown) and the miRNA-mRNA double-stranded RNA complex is blocked at the entrance to the ribosome (blue spherical complex on mRNA stand) and the miRNA-mRNA complex is degraded; the major mode of miRNA action in the mammalian brain is pathologically up-regulated miRNAs driving the down-regulation of AD-relevant genes (see text); single miRNAs can target multiple mRNAs and multiple miRNAs can target a single mRNA (see also Figure 3); miRNAs have established roles in recognizing multiple mRNA sequences (genetic pleiotropy), combinatorial and cooperativity in gene regulation, template accessibility (mediated by various RNA binding proteins; in this diagram orange spheres at the miRNA-mRNA interface called “*Argonaute proteins*”) and post-transcriptional regulation of the transcriptome (Hobert, 2008; Jaber et al., 2019; Eisen et al., 2020; Lukiw, 2020a,b). Combined with other metrics, the precise quantitation of miRNA abundance, speciation and complexity in various AD biofluids has strong potential for increasing the accuracy of AD diagnostics; recent preliminary *in vitro* studies further indicate that anti-miRNA (antimiR, antagomir, AM)-based therapies may be effective in quenching the excessive miRNA-mediated downregulation of critical mRNA-driven gene expression in AD (Zhao et al., 2016a,b; Jaber et al., 2019; Fan et al., 2020; Ghaffari et al., 2020).

microRNAs (miRNA) represent a class of ∼19–23 nucleotide (nt) single-stranded non-coding RNA (sncRNA) that are important epigenetic, posttranscriptional regulators of messenger RNA (mRNA) complexity. Their diminutive size, amphipathic nature, high solubility make them extremely mobile, omnipresent throughout the brain and CNS and the smallest information-carrying nucleic acid signaling molecules in eukaryotes yet described (Hill et al., 2014a,b; Pogue et al., 2014; Lemcke and David, 2018; Zhao et al., 2018; Lukiw, 2020a,b; van den Berg et al., 2020). To date about ∼2650 individual human miRNAs have been cataloged and characterized, however, the abundance of miRNAs in the human brain and retina number only about ∼20–35 individual, high abundance, neurologically functional species which exhibit both tissue and cell type-specific expression patterns (Burmistrova et al., 2007; Lukiw, 2007; Hill and Lukiw, 2016; Konovalova et al., 2019; Singh and Yadav, 2020; Wu and Kuo, 2020; miRBase release 22.1; October 2018^6^; last accessed 26 August 2020). The major mode of action of these sncRNAs is to interact, via base-pair complementarity, with the 3’-untranslated region (3’-UTR) of their target messenger RNAs (mRNAs), and in doing so degrade that mRNA, and hence decrease the potential for that specific mRNA to be expressed (Roshan et al., 2009; De Smaele et al., 2010; McGeary et al., 2019; Eisen et al., 2020; Pawlica et al., 2020). The most widely observed mechanism in the mammalian brain is for an inducible and up-regulated miRNA to down-regulate their polyA^+^ mRNA targets and thereby reduce the expression of mRNA-encoded genetic information. The mechanism of action of miRNAs has been described and schematized in some detail (see Hobert, 2008; Eichhorn et al., 2014; Kleaveland et al., 2018; McGeary et al., 2019; Eisen et al., 2020; Figure 2).

Interestingly, single mRNA 3’-UTRs in the mammalian brain and retina can have multiple miRNA binding site targets, and single miRNAs may have more than one mRNA target making them strong potential candidates for addressing the multiple complexities of the disruption of mRNA-based gene expression in AD (Colangelo et al., 2002; Lukiw and Alexandrov, 2012; Eichhorn et al., 2014; Eisen et al., 2020). These findings support the strengthening contention: (i) that brain-enriched miRNAs operate as fundamental components of an epigenetically controlled post-transcriptional signaling network in the mammalian CNS (Lukiw and Alexandrov, 2012; Kleaveland et al., 2018; Jaber et al., 2019; Eisen et al., 2020); and (ii) that miRNAs have an established capability to act independently, coordinately and/or cooperatively to create a highly sophisticated and interactive regulatory miRNA-mRNA network for families of brain genes that regulate many essential brain functions that are specifically altered in AD brain (Jaber et al., 2017; Kleaveland et al., 2018; Eisen et al., 2020; Lukiw, 2020a,b). Importantly, information-carrying ribonucleic acids such as highly soluble and mobile, single-stranded non-coding RNAs (sncRNAs) including microRNAs (miRNAs) can affect the operation of a large number of highly interactive pathogenic signaling pathways in the CNS, and represent strategic candidates for promoting AD onset, and for modulating or maintaining AD propagation and disease spread (Alexandrov et al., 2012; Lukiw et al., 2012; Chandrasekaran and Bonchev, 2016; Clement et al., 2016; Kleaveland et al., 2018; Lemcke and David, 2018; Hill, 2019; Jaber et al., 2019; Konovalova et al., 2019; Condrat et al., 2020; Fan et al., 2020; Kou et al., 2020).

## microRNA (miRNA) Signaling in AD

The multi-system, multi-pathway and sometimes overlapping regulatory roles for potentially pathogenic miRNA gene families in the neocortex, hippocampus, the limbic system and the CNS in general make miRNA prime candidates for modulating the expression of many mRNA targets in complex, progressive and ultimately lethal neurological disorders of the CNS that include AD (Lukiw, 2007; Zhao et al., 2016a,b; Jaber et al., 2017, 2019; Figure 2). It is for this reason there has been much recent interest in vesicle-encapsulated and/or biofluid-enriched monomeric miRNAs as potential ribonucleic acid indicators which are predictive and/or diagnostic biomarkers for the onset and development of AD (Bahlakeh et al., 2020; Krammes et al., 2020; Serpente et al., 2020; Wang et al., 2020). Indeed homeostatic levels of all 2650 miRNAs are an excellent indicator of normal brain operation and of homeostatic brain function in health and in aging, the development of dyshomeostasis, and the onset of disease. The nature of miRNA’s epigenetic and post-transcriptional regulation of about ∼27,000 messenger RNA (mRNAs), participation in a complex miRNA-mRNA linked network defining the brain cell’s transcriptome, and the shaping of this transcriptome over time under many neurophysiological conditions is both highly informative in understanding the molecular-genetics of human brain function and has been useful in the field of diagnostic and prognostic biomarkers for AD and other progressive inflammatory neurodegeneration of the human CNS (Jaber et al., 2017, 2019; Sims et al., 2020). Patterns of miRNA expression are complex – for example natural miRNA abundance has been shown to fluctuate during neural development and differentiation of the human brain and in the aging CNS (Giorgi Silveira et al., 2020; Ma et al., 2020; Wu and Kuo, 2020). Emerging data continue to support the concept that the analysis and characterization of specific miRNAs may be especially useful in the prodromal and pre-clinical phases of AD in which a very subtle pro-inflammatory neuropathology develops and molecular changes begin to accumulate even in the absence of the full-blown clinical symptoms as shown by the moderate and more advanced phases of AD (Hill and Lukiw, 2016; Bahlakeh et al., 2020; Fan et al., 2020; Ma et al., 2020; Wu and Kuo, 2020).

## microRNA (miRNA) and Progressive Inflammatory Neurodegeneration

In general, up-regulated miRNAs and down-regulation of essential neural signaling components (via down-regulation of key mRNAs) have been proposed by many independent groups to be a highly active process in the initiation and propagation of progressive inflammatory neurodegenerative diseases such as AD, amyotrophic lateral sclerosis (ALS), Huntington’s disease (HD), Parkinson’s disease (PD), trisomy 21 (T21; DS; Down’s syndrome), motor neuron disease, neurovascular disease, prion disease and several other terminal neuropathies (Holohan et al., 2013; Christoforidou et al., 2020; Singh and Yadav, 2020; Wang and Zhang, 2020; Wu and Kuo, 2020). It is interesting that to date no single newly generated *de novo* miRNA has been associated with AD – that is, miRNA alterations in AD reflect significant and absolute differences in abundance, speciation and perhaps stoichiometric relationships of existing miRNA species. Put another way, no specific miRNA “suddenly appears” at the onset, or propagation of AD, and it is a matter of up-regulation or down-regulation of an already existing miRNA species in a specific anatomical region that has been the most consistently observed in the AD brain. Interestingly, very recent molecular-genetic studies have indicated that even when derived from cell and tissue sources that are homogenous, such as pluripotent stem cells, individual cells in these unique populations often exhibit significant differences in miRNA abundance and complexity, gene expression, protein abundance and phenotypic output; here individual families of miRNAs appear to have a deterministic role in reconfiguring the *“pluripotency network”* and miRNA-mRNA linking patterns in individual cells with important downstream functional consequences (Li et al., 2015; Liu et al., 2015; Atlasi et al., 2020; Chakraborty et al., 2020; Kumar and Reddy, 2020).

Since a general down-regulation in gene expression in AD brain, especially for AD relevant components such as synaptic and cyto-architectural elements, deficits in the clearance of pro-inflammatory components and amyloid aggregates, and the consistent catabolic nature of the neurodegenerative disease process, has been repeatedly reported by multiple independent research groups, it follows that miRNAs that control gene expression in various AD tissues and biofluids could be indicative for AD-type change and perhaps diagnostic for prodromal aspects of the AD. Further, understanding of miRNA biogenesis and the signaling pathways in which groups of miRNAs participate either cooperatively or synergistically might aid the discovery of diagnostic biomarkers or development of effective therapeutics for progressive and lethal neurodegenerative disorders (Zhao et al., 2016a,b; Cole and Seabrook, 2020).

## Overview of miRNA Abundance in AD Tissues and Biofluid Compartments

One important limitation of the analysis of miRNAs in human CNS tissues, extracellular fluid (ECF) and CSF is that, apart from CNS biopsies, brain tissue samples must be obtained post-mortem, and miRNAs have a relatively short post-mortem half-life in both human brain and retina, on the range of about ∼1 to 3 h for a typical 22 nucleotide (nt) single–stranded miRNA (∼45% G + T) in the human neocortical and hippocampal compartments that have been analyzed and for which there is experimental data (Sethi and Lukiw, 2009; Rüegger and Großhans, 2012; Pogue et al., 2014; Tudek et al., 2019). Very few studies have addressed miRNA half-life *in vitro* or *in vivo* but currently both miRNA and mRNA decay kinetics have been shown: (i) to follow the same AU-enrichment rules of single-stranded miRNA and mRNA stability that is, the more AU-enriched elements (AREs) in the sncRNA, miRNA or mRNA, the shorter the half-life (Sethi and Lukiw, 2009; Rüegger and Großhans, 2012; Clement et al., 2016; Van Meter et al., 2020); (ii) to be stabilized in part by miRNA binding proteins (Zang et al., 2020); (iii) to be further stabilized by circularization (circRNA; Lukiw, 2013a,b; Zhao et al., 2016a,b; Xie et al., 2017; Kondo et al., 2020) and/or (iv) by their inclusion into exosomes or intracellular or extracellular micro-vesicles (Badhwar and Haqqani, 2020; Bitetto and Di Fonzo, 2020; Groot and Lee, 2020; Upadhya et al., 2020). Another indication of the usefulness of post-mortem material for molecular-genetic studies is that nuclei extracted from human brain biopsies or post-mortem brain tissues are able to fully support *in vitro* run-on transcription for up to ∼3–4 h after which there is a precipitous decline in polymerization activity (Cui et al., 2005; Rüegger and Großhans, 2012; Clement et al., 2016). It is important to appreciate the fact that microRNAs (miRNAs) with a mass of just ∼7628 Da (for a typical 22 nt sncRNA like miRNA-146a) are *the smallest and most abundant ribonucleic acid information-carrying components of tissues*, the ECF, the CSF and blood serum compartments. The miRNA abundance in tissues, ECF, CSF or blood serum provides valuable insight and “current snapshot” of soluble pathogenic sncRNA biomarkers that may be diagnostic for human neurological disease types. These miRNA “*information packages*” have recently been shown to be sequestered into extracellular, lipophilic microvesicles or exosomes that shuttle between cells and tissues and/or amongst ECF, CSF and blood serum compartments (Alexandrov et al., 2013; Jaber et al., 2017, 2019; Badhwar and Haqqani, 2020; Bitetto and Di Fonzo, 2020; Upadhya et al., 2020; Vanherle et al., 2020). Extracellularly secreted miRNAs circulating in the peripheral blood are referred to as “*circulating miRNAs”*; they are either encapsulated by extracellular vesicles such as exosomes and microvesicles, or bound to molecules such as the Argonaute protein, or HDL cholesterol (Zernecke et al., 2009; Arroyo et al., 2011; Vickers et al., 2011; Ishibe et al., 2018; Groot and Lee, 2020; Upadhya et al., 2020). In addition, miRNAs that leak from destroyed cells and apoptotic bodies are also found among circulating serum miRNAs and have been implicated in the spreading of AD neuropathology (Zernecke et al., 2009; Lukiw et al., 2012). It is also noteworthy to point out that while the ECF, CSF and blood serum can be considered as relatively contiguous biofluids, there may be selective biophysical barrier-mediated effects on miRNA permeability, translocation and *trans-*membrane transport of microRNAs which ends up as having the ECF, CSF, and blood serum compartments essentially distinct in their miRNA content and stoichiometric abundance (Blennow et al., 2010; Alexandrov et al., 2013; Hampel et al., 2018a,b,c; Ishibe et al., 2018; see below).

As the name suggests ECFs have been shown as being representative of the saline-based biofluids surrounding individual brain cell types (Alexandrov et al., 2012; Pogue et al., 2014; Shetty and Zanirati, 2020). ECF, sometimes referred to as interstitial fluid (ISF), drains through very narrow intercellular spaces within gray matter into bulk flow perivascular channels that surround penetrating arteries and then flows to the surface of the brain to join the CSF that drains into cervical lymph nodes (Shetty and Zanirati, 2020; Upadhya et al., 2020; Weller, 2020). Human brain CSF, produced by the choroid plexus and secreted into the brain ventricles and subarachnoid space, plays critical roles in the biophysical and immune protection of the brain and provides intra-cerebral transport of nutrients, cofactors and hormones, as well as small signaling molecules such as sncRNAs and miRNAs. Since ECF and CSF circulates throughout and within the entire CNS: (i) ECF and CSF composition is representative of the biofluids surrounding both brain cells and multiple anatomical regions of the brain and spinal cord; and (ii) provides valuable insight into soluble pathogenic bio-markers that bathe CNS cells and tissues, including soluble miRNAs that have diagnostic value for human brain health, disease or injury (Hampel et al., 2018a,b,c; Shetty and Zanirati, 2020; Weller, 2020). Human peripheral blood serum is defined as the clear-yellowish fluid that remains from blood plasma after clotting factors (such as fibrinogen and prothrombin) have been removed after clot formation and whole blood centrifugation, and contains the same components as plasma such as fatty acids, hormones, cytokines, chemokines, carbohydrates, growth factors, and miRNAs (both free miRNAs and those packaged into extracellular vesicles) and is the circulating carrier of exogenous and endogenous fatty acids, free lipids and lipoproteins in the systemic circulation (Hill, 2019; Penner et al., 2019; Lukiw and Pogue, 2020; Shetty and Zanirati, 2020^7^; last accessed 26 August 2020). The ease and relative non-invasiveness of blood serum and CSF accessibility, and acquisition in large human populations, makes it one of the most studied of all biofluid compartments in diagnostic medicine for AD and other neurological disorders.

Regarding the presence and persistence of the same miRNAs in multiple AD brain tissues and biofluids, we are aware of only one relevant peer-reviewed research report concerning miRNAs in the extracellular fluid (ECF) obtained from highly purified AD brain tissue supernatants, and parallel studies on encapsulated miRNAs packaged into extracellular vesicles in ECF and CSF (Alexandrov et al., 2012; Lukiw and Pogue, 2020). The purpose of the Alexandrov et al. (2012) paper was to ascertain if the increased miRNAs found in AD brain tissues were contiguous with up-regulated miRNAs found in AD ECF and CSF. Based on extensive fluorescent miRNA-array and RNA sequencing analysis, the results indicated significant common increases in miRNA-9, miRNA-34a, miRNA-125b, miRNA-146a, miRNA-155 that were shared by AD brain tissues, ECF and CSF. Transcription from each of these inducible miRNA genes are known to be under NF-kB (p50/p65)-regulated genetic control (Zhao et al., 2015; Lukiw, 2020a,b). Interestingly in miRNA abundance analysis of Aβ-peptide stressed human neuronal-glial (HNG) cell primary co-cultures, ECF displayed an up-regulation of these same miRNAs, an effect that was quenched using anti-NF-kB agents CAPE and CAY10512. Overall the results indicated that these same microRNAs including miRNA-9, miRNA-34a, miRNA-125b, miRNA-146a, and miRNA-155 are brain tissue-, CSF- and ECF-abundant, NF-kB-sensitive pro-inflammatory miRNAs, and their enrichment in both tissues and circulating AD biofluids suggest that they may be involved in the modulation or proliferation of miRNA-triggered pathogenic signaling throughout the human brain and CNS (Alexandrov et al., 2012; Hill, 2019; Lukiw and Pogue, 2020; Shetty and Zanirati, 2020).

In related studies and using different sources of AD samples including blood serum, post-mortem brain tissues, AD fibroblasts, AD β-lymphocytes, AD cell lines, transgenic AD (TgAD) mouse models and AD CSF all confirmed the increased presence (and biomarker potential) of miRNA-455-3p. This miRNA was found to reduce Aβ peptide toxicity, while enhancing mitochondrial biogenesis and synaptic activity and maintaining healthy mitochondrial dynamics (Kumar and Reddy, 2019; Kumar et al., 2019; Kumar and Reddy, 2020). These findings further underscore the concept that these independently documented differences may: (i) be just another example of the high levels in heterogeneity of miRNA expression in different human populations from different AD brains and patients; (ii) serve as an example of the considerable miRNA redundancy and complexity in the AD process; and (iii) indicate that multiple miRNAs are involved in the regulation of multiple gene expression pathways and patterns whose abundances are highly sensitive to alterations in the biochemical, neurochemical, neuropathological, and/or cellular environment (Alexandrov et al., 2012; Lukiw, 2013a,b; Kumar and Reddy, 2019; Kumar et al., 2019; Kumar and Reddy, 2020; Lukiw and Pogue, 2020).

## Informative miRNAS in AD

As previously discussed, the decidedly heterogeneous nature of AD appears to pervade through all molecular, genetic and epigenetic, neuropathological and behavioral, mnemonic and cognitive aspects of the disease, including the pre-clinical symptomology, initiation, disease course and presentation. Indeed in depth summaries of hundreds of peer-reviewed scientific reports to date has provided no general consensus of what single specific miRNAs are up-or-down regulated in any tissue or biofluid compartment in many thousands of AD patients, but rather a “*trend*” or “*pattern”* in certain miRNA abundance and speciation. This is perhaps not too surprising because of the manifold symptoms typically presented by AD patients, their often complicated and non-uniform drug history, the insidious nature and progressiveness of the disease, the age and gender of the AD patient, and other disease-related aspects including inter-current illness and the genetic make-up of individual AD patients.

Concerning the potential for contribution of specific miRNAs to AD, we recently surveyed the number of published papers on *“miRNA biomarkers for AD”* using the National Institutes of Health National Library of Medicine website MedLine at PubMed Central (^8^ last accessed 26 August 2020). Using the keywords “*Alzheimer’s disease’ and ‘miRNA”*) indicates that there are just under ∼13,000 publications on this topic since the original publication on selective miRNA alterations in AD brain about ∼14 years ago (Lukiw, 2007). Inspection of several of these recent reports continues to support the contention of extensive miRNA heterogeneity in both AD tissues and biofluids (ECF, CSF, and blood serum) and continues to *provide no general consensus of any single miRNA that defines causation for the onset or duration of the AD pathophysiology* (Moradifard et al., 2018; Peña-Bautista et al., 2019; Silvestro et al., 2019; Swarbrick et al., 2019; Condrat et al., 2020; Giorgi Silveira et al., 2020; van den Berg et al., 2020). It is becoming clear, however, that a panel of multiple AD-relevant “*pro-inflammatory”* or “*pro-pathology”* miRNAs may be useful in contributing to the diagnosis of AD at any stage of the disease. For example one extremely thorough systematic review was recently conducted to quantify significantly deregulated miRNAs in the peripheral blood of AD patients, and these deregulated miRNAs were cross-referenced against the miRNAs known to be deregulated in limbic regions of brain tissues, such as the hippocampus, in moderate-to-advanced stages of AD (Swarbrick et al., 2019). These analyses resulted in a panel of at least 11 miRNAs (in numerical ascendancy) - miRNA-26b, miRNA-30e, miRNA-34a, miRNA-34c, miRNA-107, miRNA-125b, miRNA-146a, miRNA-151, miRNA-200c, miRNA-210, and hsa-miRNA-485, hypothesized to be both deregulated early in AD and up to nearly ∼20 years before the onset of any clinical symptomology. Network analysis of these 11 miRNAs indicated that they were found to be associated with the cell cycle and cell surface receptor (Wnt/β-catenin) signaling, cellular response to stress, cellular senescence, gene expression regulation, and nerve growth factor and Rho GTPase signaling. Another in depth study had previously indicated that just five miRNAs – miRNA-9, miRNA-34a, miRNA-125b, miRNA-146a, and miRNA-155 – are significantly abundant in AD neocortical brain tissues as well as the ECF and CSF and each miRNA was found to interactively contribute to the AD pathophysiology (Figure 2). Interestingly, each of these five miRNAs have been shown to contain strong binding sites for the inflammatory transcription factor NF-kB in their immediate promoters, are therefore said to be under NF-kB (p50/p65) control and are referred to as “*pro-inflammatory miRNAs”* (Pogue and Lukiw, 2018; Gong and Sun, 2020; Zamani et al., 2020). The contiguous presence of specific pro-inflammatory miRNAs in AD tissue, ECF and CSF may be the result of their *trans-*compartmental solubility or may be part of a RNA-based signaling system between neocortical brain tissues and the ECF and/or CSF that is reflective or diagnostic for the neurodegenerative disease state (Alexandrov et al., 2012; Jaber et al., 2019; Veitch et al., 2019; Wang et al., 2020).

## miRNA for Precision Medicine-Based Diagnostics and Therapeutics

Extensive demographic analysis tells us that human neurodegenerative diseases such as AD are among the fastest growing neurologically incapacitating diseases of aging human populations in Westernized societies (^9^ last accessed 26 August 2020). Despite the significant medical and scientific advances made in our understanding of the AD pathophysiological mechanisms on a global scale, there is currently no effective cure for this rapidly expanding form of age related and terminally lethal senile dementia. AD is the leading cause of dementia and globally about ∼50 million people have some form of dementia, and someone in the world develops dementia every 3 s (^10^ last accessed 26 August 2020). The repeated failures of pharmacological strategies and clinical trials over the last decade in the development of novel and efficacious treatments and disease-modifying therapeutics for AD is due in part to the fact that AD is a singularly heterogeneous disorder caused by “*human genetic and biochemical heterogeneity,”* considerably different genetic and epigenetic profiles, age, gender and lifestyle factors, prodromal and more advanced phases of the disease, environmental triggers, misdiagnosis and/or importantly, the presence of other *“inter-current illness”* (Figure 1; Lukiw, 2013a, b; Blennow and Zetterberg, 2018; Cole and Seabrook, 2020; Lewczuk et al., 2020). The majority of clinical trials have focused on the immunological modulation of amyloid-β peptide (Aβ40 and Aβ42) signaling in AD, including therapeutic drugs targeted against Aβ42 peptide accumulation, inhibition of β-secretase and γ-secretase cleavage enzymes, and anti-Aβ peptide monoclonal antibody approaches, however, in most of the clinical trials for patients with mild-to-moderate AD these drugs did not meet the expected endpoints. While Aβ peptides have long been proposed to play a central role in AD, and the Aβ pathway remains a valuable therapeutic target, the accuracy, importance, and even the correctness of the “*amyloidocentric hypothesis*” in driving AD neuropathology has been recently questioned (Ricciarelli and Fedele, 2017; Mullane and Williams, 2018; Cole and Seabrook, 2020). In addition, most AD patients are diagnosed in the middle-to-late-stages of this disorder when irreversible damage to the brain has already occurred, and the “*pre-clinical*” or “*prodromal*” phase has already passed. More recently therapeutic approaches and disease modification strategies directed against other AD-implicated pathological mechanisms, including alterations in tau signaling, the cholinergic system, anti-microbial and anti-viral approaches and hormone replacement therapies, to name a few, may provide improved clinical efficacy (Bhute et al., 2020; Habes et al., 2020; Hampel et al., 2020a, b; Iqbal et al., 2020; Zhou et al., 2020).

Using data-driven studies in the identification of the earliest signs of AD and the implementation of biomarker testing and PET and/or MRI neuroimaging during the prodromal or earliest stages of the disease is an urgent contemporary quest in AD diagnostics (Zhao et al., 2015; Fierini, 2020; Emrani et al., 2020; Habes et al., 2020). This current void may be in part filled by precise miRNA profiling of miRNA-containing biofluids along the course of AD. The use of novel molecular-genetic, multimodal neuroimaging techniques and their integration under the systems biology approach should: (i) allow clinicians and researchers to better understand miRNA-based factors involved in AD initiation and trajectory; and (ii) to deliver targeted interventions tailored to the molecular-genetic and miRNA-signaling profiles of the individual AD patient, according to the precision medicine paradigm (Hampel et al., 2016, 2017, 2018a,b,c, 2019; Lista et al., 2016; Castrillo et al., 2018; Veitch et al., 2019). Such a precision medicine-based framework is now increasingly facing the clinical and biological/genetic complexity and heterogeneity of AD. This is the field where miRNA neurobiology involving both stabilized miRNAs and anti-miRNA strategies may play a significant and to date yet unexploited role (Zhao et al., 2016a,b; Jaber et al., 2019; Ghaffari et al., 2020).

It is also clear that given the complexity of AD onset, disease course and diagnosis effective future therapeutic approaches including miRNA and anti-miRNA (antimiR, antagomir, AM) strategies: (i) will need to be concurrent and multidimensional, targeting the multiple disease pathways and neurological symptoms that will both inhibit both primary disease pathogenesis and minimize ancillary off-target effects; (ii) will be integrated with advancement in the development of specific and sensitive neuroimaging and biofluid-based diagnostic tools for miRNA and other AD-relevant biomarkers; (iii) will involve *precision medicine and individualized therapies* developed within the systems biology and systems neurophysiology approaches; and (iv) will be utilized in parallel with primary medical care for screening and in a second level diagnostic work-up for specialist diagnosis and clinical management (see below; Gurland et al., 1995; Galvin et al., 2010; Hampel et al., 2016, 2019; Lista et al., 2016; Castrillo et al., 2018; Jaber et al., 2019; Penner et al., 2019; Turner et al., 2020).

## microRNA, “Human Biochemical Individuality” and Therapeutic Strategies

If high-density Gene-chip-based microarray analysis, LED-Northern analysis and current RNA sequencing strategies are of any indication of AD variability, then there are real and significant human population differences in miRNA abundance, speciation and complexity throughout the course of AD. For example one study has demonstrated variability in miRNA abundance, speciation and complexity amongst different human populations with specific reference to AD incidence amongst Caucasians and African Americans (Lukiw, 2013a,b). There continues to be an urgent requirement to identify novel protein, proteolipid and ribonucleic acid biomarkers, advanced high-resolution MRI- and PET-based neuroimaging techniques and related diagnostic methodologies for the early detection of AD in different populations. Together these will be potentially useful as a multidimensional screening technology yielding data whose integration will allow the determination, and gauge the potential risk to develop cognitive decline and/or impending neurological disruption in AD compared to healthy aging cognitively normal individuals from different population groups.

While the characterization of miRNA in neurodegenerative disease is highly informative, this information alone cannot easily discriminate between closely related neurodegenerative conditions. Many miRNA signals appear to be non-specific biomarkers of brain cell atrophy, injury or death and inflammatory neurodegeneration, response to psychoactive medications or they may be associated with other non-AD pathologies that have gone undiagnosed or misdiagnosed (Figure 1). However, in combination with other specific biomarkers or diagnostic tools, the quantification of multiple species of miRNAs might be a useful part of the diagnostic puzzle to assist in the detection and discrimination of certain specific neurodegenerative disorders even though they may possess significant clinical overlap (Juźwik et al., 2019; Kou et al., 2020; Lukiw and Pogue, 2020; Ma et al., 2020). The analysis of miRNAs over time, such as in integrated studies of neurodevelopment in humans and in experimental transgenic animal models of AD (TgAD), have already been shown: (i) to be a promising tracer and prognostic biomarker for homeostatic brain function during normal brain development and neuronal differentiation from the embryonic period to adulthood (Giorgi Silveira et al., 2020); and (ii) in conjunction with extensive neuropsychological testing, may be further useful to monitor and predict the incidence of onset, the rate of progression of disease activity and its trajectory, and to further evaluate in detail both therapeutic responses and clinical efficacy (Peña-Bautista et al., 2019). Significant heterogeneity in AD symptomology, large variation in clinical disease presentation, progression and patterns of neural system disconnection and degeneration, and molecular, genetic and epigenetic biomarkers at various stages of AD progression make this disease: (i) perhaps the preeminent example of what Linus Pauling originally referred to as “*human biochemical individuality”* – that each human individual represents a remarkably and biochemically unique case with regard to their health and susceptibility to develop disease (Pauling, 1976; reviewed in the context of AD by Lukiw, 2013a,b); and (ii) a target of both highly precise interventional treatment and the best example yet described for the potential application of “*personalized medicine*.” As previously pointed out, the strong heterogeneity in AD neuropathology, course and miRNA profiles of individuals with AD versus age- and gender matched controls strongly support this concept. The integration of multiple pharmacogenomic strategies for a personalized medical treatment in AD is now currently the most effective choice to optimize our existing therapeutic tools while reducing unwanted off-target effects. These include a significant dedication of medical personnel to individual AD patients in cooperation with family and caregivers, multiple combinatorial approaches including extensive clinical assessment, intermittent brain neuroimaging over the onset and course of AD and a host of multiple molecular, genetic, epigenetic, neurophysiological, and neurobiological strategies with focus on CSF, serum biomarkers, miRNA and perhaps other sncRNA abundance in these biofluid compartments. While significant progress is being made: it is important to point out that: (i) combinatorial diagnostic methodologies incorporating molecular genetic markers such as miRNA screening combined with DNA-based gene mutation analysis, advanced MRI- or PET-neuroimaging techniques and conscientious clinical evaluations, still have difficulty in the diagnosis of AD, often requiring the stratification of AD into complex subgroups and post-mortem verification (Guerreiro et al., 2012; Pogue and Lukiw, 2018; Penner et al., 2019; Peña-Bautista et al., 2019; Guest et al., 2020; Habes et al., 2020; Hampel et al., 2020a,b; Hudon et al., 2020; Khoury and Grossberg, 2020; Sherva et al., 2014; Rossini et al., 2020; Serpente et al., 2020; Sims et al., 2020; Singh and Yadav, 2020; Swarbrick et al., 2019; Turner et al., 2020; van den Berg et al., 2020; Wang et al., 2020; Weller, 2020); and (ii) currently available pharmacology and strategic treatments (including targeted drug delivery) based on these diagnostic tools, in the majority of cases, still do not directly address the primary underlying cause of either EOAD or LOAD but are sadly limited to the temporary alleviation of clinical symptoms (Di Resta and Ferrari, 2019; Veitch et al., 2019; Guest et al., 2020; Khoury and Grossberg, 2020; Lewczuk et al., 2020; Patnode et al., 2020; Rahman et al., 2020).

## Concluding Remarks

**FIGURE 3:**
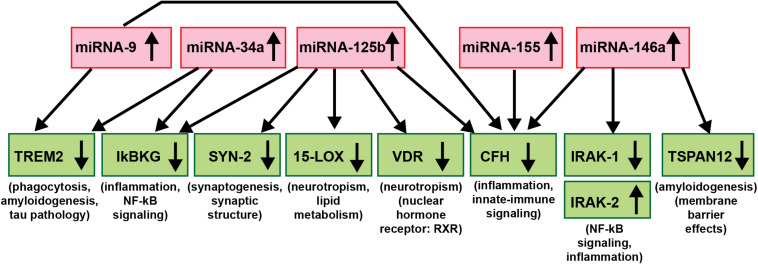
An example of a highly interactive miRNA-mRNA regulatory network involving 5 miRNAs and 9 mRNAs; there is a significant contribution of up-regulated miRNA signaling (red boxes) to specific aspects of the down-regulated mRNA-mediated expression of key AD-relevant genes (green boxes) and AD neuropathology (Chandrasekaran and Bonchev, 2016; Clement et al., 2016; Hill and Lukiw, 2016; Jaber et al., 2017, 2019; Lukiw, 2020a,b). Just 5 significantly up-regulated miRNAs – miRNA-9, miRNA-34a, miRNA-125b, miRNA-146a, and miRNA-155 (all reported to be up-regulated in AD and/or TgAD models) – can account for the down-regulation of 8 mRNA targets critically involved in multiple aspects of AD neuropathology (IRAK-2 is up-regulated due to a compensatory mechanism as described in Cui et al., 2010). Briefly, these miRNAs down-regulate miRNA-directed mRNA target degradation involved in phagocytosis deficits, amyloidogenesis and tau pathology (TREM2, TSPAN12), inflammation, NF-kB- and innate-immune signaling (IkBKG, CFH, IRAK1; with a compensatory increase in IRAK-2), neurotropism (15-LOX, VDR), synaptic maintenance and synaptogenesis (SYN-2), all of which are distinguishing pathological features characteristic of AD neuropathology. Using DNA and RNA sequencing, microfluidic-based GeneChip microarray analysis and advanced LED-Northern dot blot analysis it has been recently reported that: (i) miRNA-9, miRNA-34a, miRNA-125b, miRNA-146a and miRNA-155 are easily detected in the human brain neocortex and retina; (ii) all have NF-kB-recognition features in their immediate upstream promoters; (iii) these same miRNAs are induced by increases in NF-kB due to reactive-oxygen species (ROS) induced stress; and (iv) these 5 miRNAs form a pro-inflammatory gene family up-regulated in AD brain neocortex and hippocampal CA1 (Colangelo et al., 2002; Cogswell et al., 2008; Zhao et al., 2015; Fan et al., 2020; Lukiw, 2020a,b). This diagram is based on studies from our laboratories in which each AD and age-and gender-matched control sample (N∼135) were interrogated for 2,650 human miRNAs and 27,000 human mRNAs using RNA sequencing, microarray analysis and/or advanced LED-Northern dot blotting technologies in a single experiment and miRNA-mRNA linkage analysis and bioinformatics were subsequently analyzed (Jaber et al., 2019; Fan et al., 2020; Lukiw and Pogue, 2020; manuscript in preparation).

As critical modulators of the brain and CNS transcriptome across neurodevelopment, aging and in neurological health and disease, both in human studies and in TgAD models, research evidence continues to implicate miRNAs as major epigenetic contributors to AD onset, incidence, neuropathology, epidemiology, disease course, severity and progression (Sethi and Lukiw, 2009; Lukiw, 2013a,b; Jaber et al., 2017, 2019; Wang et al., 2017; Condrat et al., 2020; Cole and Seabrook, 2020; Lukiw and Pogue, 2020; Moradifard et al., 2018; Wang and Zhang, 2020; Wang et al., 2020; Figure 3). Importantly, to date no single miRNA has been found that is diagnostic for the “prodromal” or “MCI” phase of AD or for any particular defined stage of the disease and this is likely to remain the case in future miRNA abundance studies. The most recent findings underscore the idea that *it is very unlikely that any single miRNA in brain tissues, the ECF, CSF, blood serum, urine or any other biofluid compartments from multiple human populations will be predictive for AD at any stage of the disease.* What might be particularly useful for significantly improved AD diagnostics, however, would be a selective, high-density panel of a “*pathogenic and inflammatory neurodegeneration-associated miRNA family”* that along with other molecular-based, gene expression-based or neuroimaging-related biometrics could more accurately identify and predict the onset and course of AD-type change (Wang et al., 2017; Wang and Zhang, 2020; Wang et al., 2020; see Figure 3). According to the systems biology approach, one of the pillars of precision medicine, a comprehensive evaluation, encompassing multiple pathophysiological mechanisms at different biological levels is needed to fully untangle the dynamics of AD and inform therapeutic decision-making at the individual level. miRNA-, mRNA- and protein-based gene expression alterations and patterns, AD-relevant DNA mutations, pro-inflammatory biomarkers, such as the novel flood of inflammatory cytokines contributing to the cytokine storm in AD, Aβ40- and Aβ42-peptide load in the ECF, CSF and blood serum, combined with data from MRI- and PET-based neuroimaging, familial and clinical history and lifestyle factors could be extremely useful in improving diagnosis and prognosis of AD onset and development and perhaps, even the susceptibility to AD initiation and/or development (Zhao et al., 2015; Wang et al., 2017; Frost et al., 2019; Hampel et al., 2019; Juźwik et al., 2019; Swarbrick et al., 2019; Adams et al., 2020; Condrat et al., 2020; Hampel et al., 2020a,b; Serpente et al., 2020; Turner et al., 2020; Wang and Zhang, 2020; Wang et al., 2020).

One may argue that the heterogeneity of miRNAs in AD tissues and biofluids may make these ribonucleic acid-based biomarkers too variable in reflecting a pathological condition, however, of the known ∼2650 currently identified human miRNAs only about ∼35 miRNA species are known to be abundant in the brain, retina and CNS (see above;^11^ Lukiw, 2007; Zhao et al., 2015; Jaber et al., 2017). If miRNA abundance is any reflection of its importance, this appears to significantly restrict the total number of expressed miRNAs that may be mis-regulated in AD brain. Moreover the basis of “*individualized prevention, precise and personalized”* treatment strategies using a systems biology approach requires an input from a very large number of independent data sources. While miRNA abundance, speciation and complexity is a very important one of these data sources, it is the information that may be extracted from all of these data sources together that should be the most effective as a diagnostic, prognostic and/or screening tool across the entire continuum of AD (Wang et al., 2015, 2017; Jaber et al., 2017, 2019; Lukiw and Pogue, 2020; Wang and Zhang, 2020; Wang et al., 2020).

Lastly, multiple analytical molecular-genetic approaches, geriatric, and clinical evaluation, current neuroimaging methods and resulting integrated diagnostic and predictive strategies are currently within the capabilities of contemporary clinical and medical neurology. Improved clinical data acquisition, coordination, interpretation and integration of clinical, laboratory and healthcare resources will be required to obtain a more accurate diagnostic profile of the “*provisional AD patient*.” miRNA-mRNA linkage or association mapping for AD-relevant neurological pathways should be additionally useful as a diagnostic approach because miRNA-mediated regulatory mechanisms appear to involve a large number of pathogenic and highly integrated gene expression pathways in the CNS. An equally wide variety of “*individualized prevention, precise and personalized”* treatment strategies will also be required to more effectively address AD and other insidious, age-related neurological disorders, including the application of novel and highly customized, personalized and/or combinatorial miRNA modulatory and/or anti-miRNA-based pharmacological strategies whose therapeutic design and implementation have yet to be considered.

## Disclosure

HH is an employee of Eisai Inc. and serves as Senior Associate Editor for the Journal Alzheimer’s and Dementia and does not receive any fees or honoraria since May 2019; before May 2019 he had received lecture fees from Servier, Biogen and Roche, research grants from Pfizer, Avid, and MSD Avenir (paid to the institution), travel funding from Functional Neuromodulation, Axovant, Eli Lilly and company, Takeda and Zinfandel, GE Healthcare and Oryzon Genomics, consultancy fees from Qynapse, Jung Diagnostics, Cytox Ltd., Axovant, Anavex, Takeda and Zinfandel, GE Healthcare and Oryzon Genomics, and Functional Neuromodulation, and participated in scientific advisory boards of Functional Neuromodulation, Axovant, Eisai, Eli Lilly and company, Cytox Ltd., GE Healthcare, Takeda and Zinfandel, Oryzon Genomics and Roche Diagnostics. HH is co-inventor in the following patents as a scientific expert and has received no royalties:

*In vitro* Multiparameter Determination Method for The Diagnosis and Early Diagnosis of Neurodegenerative Disorders Patent Number: 8916388;

*In vitro* Procedure for Diagnosis and Early Diagnosis of Neurodegenerative Diseases Patent Number: 8298784;

Neurodegenerative Markers for Psychiatric Conditions Publication Number: 20120196300;

*In vitro* Multiparameter Determination Method for The Diagnosis and Early Diagnosis of Neurodegenerative Disorders Publication Number: 20100062463;

*In vitro* Method for The Diagnosis and Early Diagnosis of Neurodegenerative Disorders Publication Number: 20100035286;

*In vitro* Procedure for Diagnosis and Early Diagnosis of Neurodegenerative Diseases Publication Number: 20090263822;

*In vitro* Method for The Diagnosis of Neurodegenerative Diseases Patent Number: 7547553;

CSF Diagnostic *in Vitro* Method for Diagnosis of Dementias and Neuroinflammatory Diseases Publication Number: 20080206797;

*In vitro* Method for The Diagnosis of Neurodegenerative Diseases Publication Number: 20080199966;

Neurodegenerative Markers for Psychiatric Conditions Publication Number: 20080131921;

AV is an employee of Eisai Inc. He does not receive any fees or honoraria since November 2019. Before November 2019 he had received lecture honoraria from Roche, MagQu LLC, and Servier.

SL has received lecture honoraria from Roche and Servier.

WL serves on 36 journal editorial boards and study sections and is senior academic editor for the journals Frontiers in Genetics, Molecular Neurobiology and PLOS One. Research on miRNA and mRNA in the Lukiw laboratory involving biomarkers in AD and in other forms of neurological or retinal disease, amyloidogenesis, synaptogenesis and neuroinflammation was supported through an unrestricted grant to the LSU Eye Center from Research to Prevent Blindness (RPB); the Louisiana Biotechnology Research Network (LBRN) and NIH grants NEI EY006311, NIA AG18031 and NIA AG038834 (WL).

## Author Contributions

YZ, VJ, PA, AV, SL, HH, and WL researched the manuscript. WL assembled all data and wrote the manuscript. All authors contributed to the article and approved the submitted version.

## Conflict of Interest

The authors declare that the research was conducted in the absence of any commercial or financial relationships that could be construed as a potential conflict of interest.

## Footnotes

1 https://www.alzinfo.org/articles/when-patients-with-advanced-alzheimers-get-pneumonia/

2 https://www.medicalnewstoday.com/articles/315123

3 https://www.alz.org/alzheimers-dementia/stages

4 https://www.j-alz.com/editors-blog/posts/is-there-alzheimers-disease

5 https://knightadrc.wustl.edu/cdr/cdr.htm

6 www.mirbase.org

7 https://www.innov-research.com/blogs/news/the-importance-of-human-serum

8 www.ncbi.nlm.nih.gov

9 https://www.alz.org/alzheimers-dementia/facts-figures

10 https://www.usagainstalzheimers.org/learn/alzheimers-crisis?gclid=EAIaIQobChMI287JsPG96QIVD dbACh0HjAm0EAAYBCAAEgILDvDBwE

11 http://www.mirbase.org/

## Abbreviations

AD: Alzheimer’s disease
AD: biomarkers
AD: diagnostics
AD: heterogeneity
human biochemical individuality
inflammatory neurodegeneration
microRNA (miRNA).
